# Addressing Diagnosis, Management, and Complication Challenges in Placenta Accreta Spectrum Disorder: A Descriptive Study

**DOI:** 10.3390/jcm13113155

**Published:** 2024-05-28

**Authors:** Marfy Abousifein, Anna Shishkina, Nicholas Leyland

**Affiliations:** 1 Health Sciences Department, McMaster University, Hamilton, ON L8S 4L8, Canada; 2 McMaster University Medical Center, Hamilton, ON L8N 3Z5, Canada

**Keywords:** obstetrics/gynecology, neonatology, women’s health, rural health

## Abstract

Introduction: In light of increased cesarean section rates, the incidence of placenta accreta spectrum (PAS) disorder is increasing. Despite the establishment of clinical practice guidelines offering recommendations for early and effective PAS diagnosis and treatment, antepartum diagnosis of PAS remains a challenge. This ultimately risks poor mental health and poor physical maternal and neonatal health outcomes. Case Descriptions: This case series details the experience of two high-risk patients who remained undiagnosed for PAS until they presented with antenatal hemorrhage, leading ultimately to necessary, complex surgical interventions, which can only be optimally provide in a tertiary care center. Patient 1 is a 37-year-old woman with a history of three cesarean sections, which elevates her risk for PAS. She had placenta previa detected at 19 weeks, and placenta percreta diagnosed upon hemorrhage. During a hysterectomy, invasive placenta was found in the patient’s bladder, leading to a cystotomy and right ureteric reimplantation. After discharge, she was diagnosed with a vesicovaginal fistula, and is currently awaiting surgical repair. Patient 2 is a 34-year-old woman with two previous cesarean sections. The patient had complete placenta previa detected at 19- and 32-week gestation scans. She presented with antepartum hemorrhage at 35 weeks and 2 days. An ultrasound showed thin myometrium at the scar site with significant vascularity. A hysterectomy was performed due to placental attachment issues, with significant blood loss. Both patients were at high risk for PAS based on past medical history, risk factors, and pathognomonic imaging findings. Discussion: We highlight the importance of the implementation of clinical guidelines at non-tertiary healthcare centers. We offer clinical-guideline-informed recommendations for radiologists and antenatal care providers to promote early PAS diagnosis and, ultimately, better patient and neonatal outcomes through increased access to adequate care.

## 1. Introduction

Placenta accreta spectrum disorder (PAS) is a pregnancy complication describing the abnormal adherence of the placenta, leading to an increased risk of maternal and neonatal morbidity and mortality [1,2,3,4]. Normally, the placenta attaches to the endometrium, which is the innermost layer of the uterus and separates from the uterine wall, leading to easy detachment and subsequent delivery [1]. However, in PAS, the placenta can invade the deeper layers of the uterus, including the myometrium (middle layer) or even the serosa (outer layer) and contiguous structures. When the placenta has formed these abnormal attachments, it cannot detach properly after the delivery of the baby. Thus, the risk of complications during delivery increases, as spontaneous or forced placental separation can lead to massive hemorrhage [2,3,4]. PAS classification includes the following grades: Grade 1—abnormally adherent placenta (placenta adherenta or creta); Grade 2—abnormally invasive placenta (Increta); Grade 3—abnormally invasive placenta (percreta). Grade 3 includes the following sub-grades: Grade 3a—invasion limited to uterine serosa; Grade 3b—urinary bladder invasion; Grade 3c—invasion of other pelvic tissue/organs [4].

When early PAS diagnosis is made based on skilled available imaging, patients undergo counseling and frequent antenatal visits for close monitoring by a specialized multidisciplinary team [5]. Cesarean hysterectomy, with the placenta in situ, is generally agreed to be the preferred approach to management [6]. Cell salvage and devascularization methods, as well as enough blood products, are essential to providing necessary and patient-centered PAS treatment. Conservative management, with the goal of preserving fertility, may be considered in very select cases with careful follow-up [7].

Current estimates suggest a PAS incidence of 1 in 500 pregnancies in middle- and high-income countries, a ten-fold increase over the last 40 years, following a significant increase in the rate of cesarean section [2,8,9,10]. PAS is associated with significant maternal morbidity and mortality, secondary to massive maternal hemorrhage when separating or delivering the placenta [1,2,3,9]. With the increasing incidences and the severity of the condition, it is essential that non-tertiary healthcare centers are able to diagnose PAS, allowing referral to appropriate care centers where the needed resources and specialists are available and for the patient to undergo counselling. 

Here, we present two cases of high-risk patients who presented with antenatal hemorrhage in the third trimester and, upon further investigations, were diagnosed with PAS. Delayed diagnosis led to life-threatening complications, which could have been avoided with earlier PAS diagnosis. This case series is unique in that it uses these cases of systemic failures to make recommendations for community hospital practitioners and antenatal providers outside of tertiary-care centers to promote early PAS intervention and lead to increased access to adequate care and, by implication, better health outcomes.

## 2. Case 1: History, Investigations, Treatment, and Outcomes

The first patient is a 37-year-old female (G4P3) with a history of three previous cesarean sections. An anatomy scan at 19 weeks’ gestation showed a complete placenta previa. An ultrasound performed at 33 weeks of gestation identified anterior placenta previa with multiple placental lakes. Despite the patient’s high-risk medical history and the pathognomonic ultrasound findings, no further investigation was conducted for the possibility of PAS, and she was scheduled for a repeat elective cesarean section at a community hospital. At 34 + 4 weeks’ gestation, she presented with antenatal hemorrhage and was referred to our tertiary center from a community hospital for suspected placenta percreta. An ultrasound at 34 weeks and 4 days of gestation at our center revealed placenta percreta in the anterior midline and left aspects. Therefore, she consented to a hysterectomy at the time of cesarean section. At the time of the hysterectomy, invasive placentation was noted in the posterior bladder at the level of the cervix. Despite meticulous surgical technique by our experienced surgical team, a cystotomy was performed in order to perform a complete resection of the placenta percreta. Given the cystotomy location in intimate proximity to the bladder trigone and, in particular, the right ureteric orifice, the urology team performed right ureteric reimplantation and ureteric stenting. Intraoperative blood loss was three liters, and she received four units of packed cells, three units of packed cells from cell-saver, as well as colloids and other blood products. She recovered well in the hospital and was discharged home with an indwelling catheter. Five weeks post-operation, she was diagnosed with a one-centimeter vesicovaginal fistula and is currently awaiting surgical repair. The fetus was in transverse presentation and had a normal biophysical profile. There were no complications with the fetus. 

## 3. Case 2: History, Investigations, Treatment, and Outcomes

**Figure 1:**
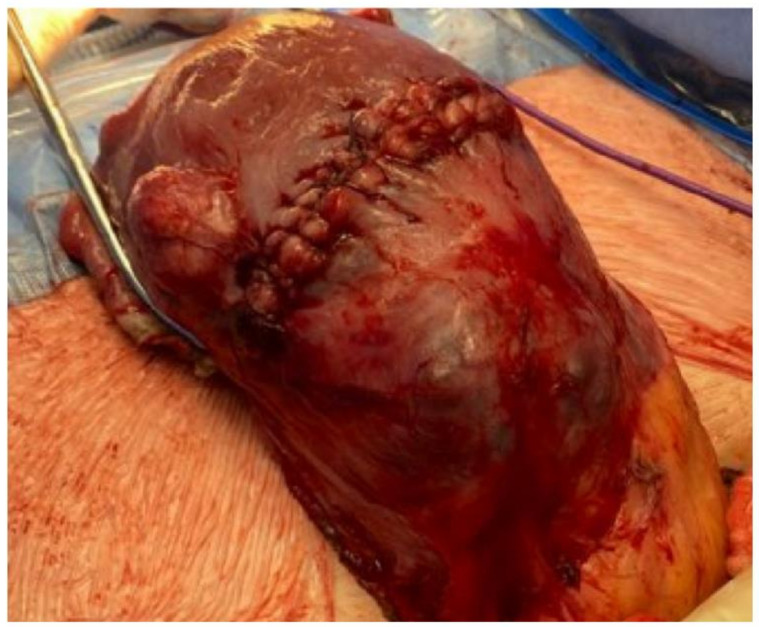
Uterus with visible placenta accreta.

The second patient is a 34-year-old female (G4P2A1) with two previous caesarian sections and a known posterior complete placenta previa in this pregnancy seen at the 19- and 32-week gestation anatomy scans. She presented with antepartum hemorrhage at 35 weeks and 2 days of gestation. An ultrasound at our center showed myometrium < 1 mm at the level of the previous cesarean section scar and significant vascularity. She consented to a possible hysterectomy at the time of cesarean section if there would not be spontaneous placental detachment. Intraoperatively, it was found that the placenta was bulging through the myometrium and was covered with a serosal layer only (Figure 1). Although a hysterectomy was completed with bilateral internal iliac artery ligation, surgical blood loss was four and a half liters, and she required a transfusion of six pRBC units as well as other blood factors. Her hospital stay and recovery were uneventful. Fetal presentation was cephalic, and they had a normal biophysical profile. There were no complications with the fetus. 

## 4. Discussion

### 4.1. Diagnostic Comments

To assess the possibility of PAS, and make a diagnosis, providers consider risk factors, clinical presentations, and histological findings. Risk factors for PAS include placenta previa, multiple cesarean sections, uterine surgeries (e.g., myomectomy, operative hysteroscopic procedures, dilation, and curettage, etc.), advanced maternal age, smoking, multiple births, multiple abortions, and hypertensive pregnancy disorders of pregnancy [11,12,13]. The incidence of PAS increases with the number of previous cesarean deliveries, ranging from 0.3% in patients with one prior cesarean section to approximately 7% for those with more than five cesarean deliveries [12,14]. Among pregnant women diagnosed with placenta previa, the frequency of PAS notably increases with the number of cesarean deliveries (3%, 11%, 40%, 61%, and 67% for the first, second, third, fourth, and fifth or more cesarean births, respectively) [12,15]. Advanced maternal age increases the risk of placenta previa and previous uterine surgery, thereby increasing PAS risk [12,16]. Some cases of PAS have been reported without relevant prior history, but with the presence of uterine pathologies such as adenomyosis, submucous fibroids, bicornuate uterus, or basal plate myometrial fibers observed in the placenta post-delivery [12,17,18,19]. These pathological factors were suggested without sufficient high-quality evidence, highlighting the need for further studies. 

Typically, the first clinical presentation of PAS is obstetric hemorrhage with attempts to remove the placenta during delivery [12]. In those with placenta previa, antenatal bleeding may also be observed [12]. Placenta recreate can cause abdominal pain due to uterine rupture in some cases [12]. In asymptomatic patients with risk factors, obstetric ultrasounds should arouse PAS suspicion [12]. Antenatal diagnosis is important to help ensure the patient undergoes proper counselling and PAS management, as well as decrease the chance of experiencing great blood loss [12]. Unfortunately, “studies showed that approximately one-half to two-thirds of cases are not diagnosed antenatally demonstrating the necessity of a targeted prenatal screening for PAS with appropriate timing and candidates” [12,16,20,21].

The earliest signs of PAS in ultrasound may occur in the first trimester: PAS should be suspected if a gestational sac is found near the lower uterine segment and near or lower than the cesarean scar during the first-trimester ultrasound [22,23]. Yet, according to the literature, the majority of these patients are usually diagnosed later on in their pregnancy due to placenta previa [12,24,25,26].

For high-risk patients, ultrasonography, between 18 and 24 weeks of gestation, on the interface between the placenta and myometrium (transabdominal and transvaginal) should be evaluated by a multidisciplinary team (including experienced sonographers and obstetricians) [12,27]. This timing is ideal as the diagnosis of PAS can be confirmed or excluded with up to 90% of pregnant people [12,16,20,21]. According to Liu et al., there is very limited evidence regarding the schedule for ultrasound examinations among pregnant people without specific risk factors of PAS [12]. Thus, more studies are needed to guide clinical practice.

MRI has advantages over ultrasound when a PAS occurs in a posterior position as the bladder cannot be used to identify the placental–myometrial interface [12,27,28]. MRI can better evaluate the invasive depth of PAS, myometrial, and parametrial involvement, and bladder involvement relative to ultrasonography. This can be best performed between 24 and 30 weeks of gestation because false positive and negative rates are significantly higher beyond this period [12,29]. However, more studies are needed to confirm this guidance. 

### 4.2. Management Comments

The goal is to plan a cesarean section during a time in gestation, optimizing neonatal outcomes and minimizing maternal risks. While the literature is unclear on the most optimal timing for cesarean section, there is a higher risk for hemorrhage past 36 weeks of gestation; thus, deliveries are planned for 34 weeks of gestation [12,24,30]. Based on patient stability, the date of delivery can be planned closer to or further from the 36-week gestation mark. Preeclampsia, membrane rupture, hemorrhage, fetal compromise, or progressive maternal comorbidities should push up the date of cesarean section [12,30].

It is essential to address anemias and optimize hemoglobin levels, given the chance of hemorrhage accompanying PAS [12]. Between 23 and 34 weeks, consider corticosteroid treatment for patients experiencing antenatal bleeding [31,32]. For those living far away from tertiary care centers and experiencing bleeding, consider hospitalization during late pregnancy [12]. The use of drugs to prevent bleeding, such as tranexamic acid, remains uncertain and under investigation [33].

## 5. Recommendations 

To the best of our knowledge, there is a notable gap in the literature addressing the systemic shortcomings of PAS management in non-tertiary healthcare centers. We conducted a literature review of English studies using PUBMED, MEDLINE, and CINHAL. Research terms included “placenta accreta”, “non-tertiary”, and “management”. Only one study was found discussing the implementation of multidisciplinary models of care in community hospitals; however, no concrete recommendations are provided for non-tertiary healthcare center practitioners [34]. While there is a lack of research on diagnosing and managing PAS in non-tertiary care centers, the cases we present here, which went undiagnosed until complications, are not a few isolated incidents. Instead, these cases are indicative of common systemic failures that are endangering maternal and neonatal health, particularly for patients in rural areas. Both cases involve delayed PAS diagnosis in patients with high-risk factors for PAS, such as a history of previous cesarean sections and associated pathognomonic ultrasound findings, such as placenta previa, which should have led to further investigation and, ultimately, early PAS diagnosis. These diagnostic delays led to life-threatening hemorrhages, necessitating the use of cell salvage, extensive blood transfusions, as well as surgical complications needing the expertise of multiple specialists. Such diagnostic delays, therefore, lead to increased healthcare costs and compromised patient mental health due to the traumatic experience of suffering PAS complications without having received prior counselling or diagnosis. Additionally, having to travel distances for emergency access to care at tertiary centers, patients from rural areas may face worse mental health outcomes due to the lack of familial or community support and financial strain. Further, due to delays as a result of long travel distances, patients are at risk for worse health outcomes. This is a risk that can further compromise patient mental health. This will disproportionately impact rural and low-income patients, highlighting health disparities and barriers to accessing adequate healthcare for some patient populations. Considering that patients with placenta percreta and those undergoing emergency surgical intervention have a high risk of urological complications further displays the need of early diagnosis to allow the transfer of patients to medical centers equipped for such complications [35]. 

Additionally, receiving a late PAS diagnosis prevents patients and their families from receiving the necessary counselling. This not only compromises patient mental health, but also deprives the patients of being able to make decisions regarding their reproductive health within an appropriate amount of time and with more options for PAS management. This is because, when a PAS diagnosis is made due to severe complications, such as hemorrhage, immediate care must be received, which limits the time the patients have to make healthcare decisions. Moreover, during counselling, patients and their providers will be able to make a plan regarding the mode of delivery (cesarean or vaginal), whether to remove the placenta at the time of delivery, therapies to prevent hemorrhage, and the type of hysterectomy (total or subtotal), if needed. When patients do not receive an antenatal diagnosis, and by implication the counselling, their options regarding the aspects of care described above are reduced, with some becoming impossible due to complications or associated with a greater risk of poor healthcare outcomes. 

Due to the poor consequences of diagnostic delays, this report strives to inform healthcare providers, providing them with the informational support they need to make early PAS diagnoses. 

In community and non-tertiary care centers, providing adequate treatment for cases that are suffering from complications due to late PAS diagnosis may be challenging. These centers often lack sufficient blood products for transfusions. They also lack cell salvage devices, compromising the quality of care for individuals who cannot receive blood transfusions due to religious or cultural reasons. This ultimately increases the risk of maternal morbidity and mortality. Furthermore, PAS management requires the expertise of advanced gynecology surgeons and possibly urologists, who are likely unavailable at community healthcare centers. Neonatal and maternal intensive care, as well as maternal–fetal specialists, are also not typically available outside of tertiary healthcare centers. This poses potential risks to both the pregnant person and the fetus, specifically if the patient cannot receive blood. This highlights healthcare disparities between different religious and cultural groups. Moreover, requiring immediate care for complications can also lead to receiving care at unequipped healthcare centers, as described above. This can lead to fewer treatment options for the patient and potentially eradicate the choice for conservative management that preserves fertility. This can limit the patient’s autonomy and decision-making power regarding future reproduction. 

The cases reviewed demonstrate the challenge of timely diagnosis of PAS cases, even in relatively unambiguous patients with a high risk for PAS. All centers providing care to patients at risk of PAS should follow the recommendations entailed within North American clinical guidelines, such as the following: (1) the Society of Obstetricians and Gynaecologists of Canada’s *Guideline No. 383-Screening, Diagnosis, and Management of Placenta Accreta Spectrum Disorders*; (2) the American College of Obstetrics and Gynecologists’s *Obstetrical Care Consensus: Placenta Accreta Spectrum* [2,5]. Making decisions that are informed and guided by such guidelines can help promote early PAS diagnosis, and, by implication, better healthcare outcomes and the use of fewer healthcare resources. 

In light of these challenges, we propose the following recommendations (Table 1) to educate radiologists, obstetricians, gynecologists, midwives, and family physicians, with the goal of promoting early PAS diagnosis and access to early intervention, leading to better healthcare outcomes [2,5].

Furthermore, for patients from marginalized and equity-deserving groups, such as Black and Indigenous patients, medical mistrust is a prominent issue that can act as a barrier to seeking care from obstetricians and gynecologists or seeking care at a tertiary healthcare center. As a result, many pregnant people opt to receive antenatal and obstetric care from midwives and alternative medicine services providers and to have their babies delivered at their homes. Ultimately, this enhances the barriers to antenatal PAS diagnosis and, thus, timely and adequate care. This further illustrates the need for educating antenatal service providers and raising awareness about PAS and its risk factors. Additionally, this highlights the need for culturally competent healthcare that encourages and welcomes patients from diverse backgrounds, as well as the need to diversify the healthcare provider population in order to provide adequate and equitable care to all patients. 

Additionally, according to the literature, compared to white women, Black, Hispanic, and Asian women are less likely to have private health insurance [37]. Those with public health insurance are more likely to receive prenatal care later in their pregnancy, which impacts maternal outcomes and may lead to a later diagnosis of PAS [37]. Considering how finances play a role in prenatal care and how, for uninsured patients in areas without universal healthcare, it is important that patients receive proper counselling to allow for autonomous and risk-conscious healthcare decisions. Counselling should be conscious of the financial strain associated with diagnostic assessments and specialist consultations for PAS. Patients should be advised about the risks associated with different management options. Providers should foster a welcoming, honest, and empathetic environment for discussion, allowing patients and their families to share their concerns regarding finances and how they factor into the PAS management and diagnosis process. Counselling should be culturally competent, considering that those who are uninsured are more likely to be non-white patients [37]. Such care approaches and environments are important as the literature shows that post-surgical complications include high rates of posttraumatic stress disorder, pain, decreased quality of life, and poor mental health [38].

## 6. Conclusions

In conclusion, the cases presented here underscore the systemic deficiencies in managing PAS. Diagnostic delays due to a lack of awareness and resources can lead to life-threatening complications, extensive blood loss, and surgical complications, ultimately impacting patient well-being and increasing healthcare costs. Access to adequate blood products, specialized surgical expertise, and neonatal and maternal intensive care is often limited in community healthcare centers. This poses significant risks to patients whose PAS diagnosis delays hinder their seeking of treatment at tertiary healthcare centers. We recommend adherence to established guidelines for PAS management and propose recommendations targeting healthcare providers to improve early diagnosis and intervention, thereby enhancing patient outcomes and reducing disparities in care.

## Figures and Tables

**Figure 2:**
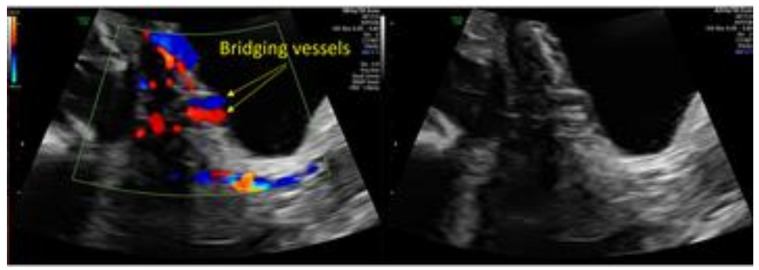
“Bridging vessels” interrupting the bladder wall in color Doppler ultrasound (**left**) and grey-scale ultrasound (**right**). Imaged adopted from Adu-Bredu et al. [36].

**Figure 3:**
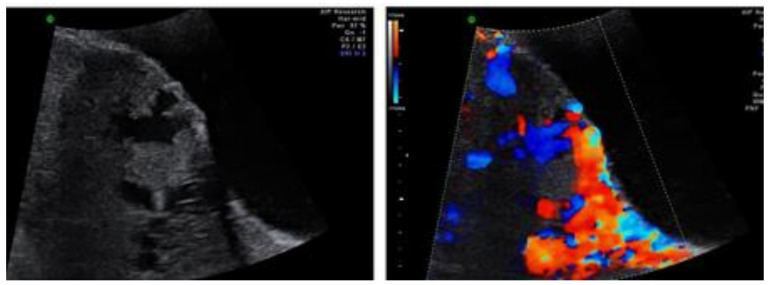
PAS lacune in color Doppler ultrasound (**right**) and grey-scale ultrasound (**left**). Imaged adopted from Adu-Bredu et al. [36].

**Table 1:** Recommendations to Promote Early PAS Diagnosis.

	Recommendations	Target Practitioner
Patients with high risk for PAS, such as maternal age ≥ 35, in vitro fertilization, cesarean section, or previous uterine surgery.	(1) Talk with patients about the risk of PAS, signs, and potential management. This discussion should be informed by culturally and religiously competent practices. Consider potential treatments that the patient is comfortable with and the cultural and religious factors that may influence health-seeking behavior. Also consider the patient’s health literacy, which may influence how they respond to health information. (2) At 18–20 weeks, request a specialist ultrasound and a radiological report on placentation. Provide radiologist with patient history.	Managing antenatal specialties such as obstetricians, gynecologists, midwives, and family physicians.
When to refer a patient to a tertiary healthcare center.	(1) Suspicion of PAS based on ultrasound findings. (2) Inconclusive ultrasound results regarding placentation. (3) The patient is at high risk for PAS and experiencing hemorrhage.	Managing antenatal specialties such as obstetricians, gynecologists, midwives, and family physicians.
When conducting a prenatal ultrasound.	(1) Request patient history to inquire about factors contributing to PAS risk. (2) Consider different PAS markers during each trimester of pregnancy. (3) Take note of the following: placenta previa, thickened placenta towards bladder wall, placental lacunae, myometrial thinning, interruption of the hyperechoic bladder serosal line, intravesical placental tissue, or hemorrhagic clot (Figure 2 and Figure 3).	Radiologists.

## Data Availability

The data presented in this study are available on request from the corresponding author. The data are not publicly available due to privacy.
